# miR-134-3p driven by anisomycin impairs ovarian cancer stem cell activity through inhibiting GPR137 expression

**DOI:** 10.7150/jca.87692

**Published:** 2023-10-16

**Authors:** Lele Ling, Yichao Wen, Haiyang Chen, Ying Xiong, Xin Liu, Juan Chen, Te Liu, Bimeng Zhang

**Affiliations:** 1 Department of Acupuncture and Moxibustion, Shanghai General Hospital, Shanghai Jiao Tong University School of Medicine, Shanghai 200086, China.; 2 Shanghai Geriatric Institute of Chinese Medicine, Shanghai University of Traditional Chinese Medicine, Shanghai 200031, China.; 3 Department of Obstetrics and Gynecology, Xinhua Hospital Affiliated to Shanghai Jiao Tong University School of Medicine, Shanghai 200092, China.; 4 Department of Dermatology, Yueyang Hospital of Integrated Traditional Chinese and Western Medicine, Shanghai University of Traditional Chinese Medicine, Shanghai 200437, China.; 5 Gongli Hospital Affiliated to the Second Military Medicical University in Pudong New Area of Shanghai City, Shanghai 200135, China.

**Keywords:** Human ovarian cancer stem cells, DLK1-DIO3 imprinted microRNA cluster, miR-134-3p, Anisomycin, GPR137

## Abstract

**Background**: Ovarian cancer recurrence and metastasis are predominantly attributed to ovarian cancer stem cells; however, the mechanism by which anisomycin regulates human ovarian cancer stem cells (HuOCSCs) remains unclear.

**Methods**: cDNA microArray was used to screen microRNAs (miRNAs) targeted by anisomycin, and RT-qPCR validated the miRNA targets. TargetScan database, GO enrichment analysis, and RT-qPCR, accompanied by a fluorescent reporter system, were employed to verify the miRNA target genes. *In vitro* experimental cell proliferation inhibition assay, flow cytometry, Transwell, angiogenesis assay, and *in vivo* transplantation tumor assay were implemented to assess the ability of the overexpressed miRNAs to hinder HuOCSC activity. Western blot, RT-qPCR, and immunofluorescence were applied to measure the transcriptional and protein-level expression of the miRNA target genes and their related genes. Bioinformatic analysis predicted and deciphered the role of the miRNA target genes and related genes in the development and prognosis of ovarian cancer.

**Results:** The expression levels of multiple DLK1-DIO3 imprinted microRNA cluster members were altered by anisomycin, among which miR-134-3p expression was most significantly elevated. miR-134-3p overexpression significantly suppressed HuOCSC activity. The screening and validation of target genes uncovered that miR-134-3p was able to markedly suppress GPR137 expression. Additionally, miR-134-3p regulated the cytoskeleton, migration-related protein in the NDEL1/DYNEIN/TUBA1A axis through targeting GPR137. Bioinformatics prediction unveiled a close association of GPR137, NDEL1, DYNC1H1, and TUBA1A with ovarian cancer development and prognosis.

**Conclusions:** The activity of HuOCSCs may be compromised by anisomycin through the regulation of miR-134-3p, which inhibits the GPR137/NDEL1/DYNEIN/TUBA1A axis.

## Introduction

Ovarian cancer critically endangers women's health as it is a gynecological tumor with an extremely high degree of malignancy 1-4. Previous studies have reported the existence of a subpopulation of embryonic stem cell-like cells in ovarian cancer tissues, which highly express CD44, CD133, and c-Kit (CD117). This cell subpopulation has stemness features of stem cells but maintains tumor cell properties such as high proliferation, invasion, and tumorigenesis; therefore, these cells are regarded as ovarian cancer tumor stem cells (HuOCSCs) 5-7. Due to high heterogeneity of OCSCs and high resistance to conventional chemotherapeutic drugs, developing drugs that can efficiently target and destroy OCSCs is urgently needed.

Anisomycin is the first validated antiprotozoal drug, and it was derived from Streptomyces griseolus. It was initially reported that anisomycin could impede protein synthesis and facilitate aging and tumorigenesis in most cells through interacting with the 60S ribosomal subunit and suppressing the formation of peptide bonds. Yu et al. discovered that anisomycin suppressed Jurkat T-cell proliferation by activating p53, p21, and p27 signaling pathways and arresting S and G2/M phases 8. Seo et al.'s study revealed that the administration of anisomycin resulted in the downregulation of Bcl-2, c-FLIP(L), and Mcl-1, ultimately leading to the induction of apoptosis in renal tumor cells 9. Further, Liu et al. demonstrated that the induction of apoptosis by anisomycin in glucocorticoid-resistant acute lymphoblastic leukemia cells was mediated through the activation of mitogen-activated protein kinase (MAPK) p38 phosphorylation and Jun amino-terminal kinase 10. Wei Chen et al.'s recent study has uncovered that anisomycin can facilitate H3S10 phosphorylation modification by driving p38 MAPK into the nucleus, which ultimately leads to ferroptosis in hepatocellular carcinoma cells 11. The above findings support that anisomycin is a potential chemotherapeutic drug.

microRNAs (miRNAs) are a group of noncoding RNAs without an open reading frame; they have between 21 and 23 nucleotides and have a secondary structure 7, 12, 13. They are widely found in eukaryotes and are highly conserved in terms of structure and sequence 7, 12, 13. Although miRNAs do not encode proteins, they can silence a target gene by binding to a specific site on the 3' noncoding region of the target gene's mRNA 7, 12, 13. Lines of studies have indicated that miRNAs participate in tumorigenesis and development. As research has progressed, attention has been drawn to the presence of DLK1-DIO3 imprinted microRNA cluster localized on distal mouse chromosome 12 and human chromosome 14, which has variable structural types 12-16. The imprinted domain comprises a triad of paternally expressed genes encoding retrotransposon-like gene 1 (Rtl1/Mart 1), delta-like homologue 1 (Dlk1), and type 3 deiodinase (Dio3), in addition to a multitude of maternally expressed imprinted noncoding RNAs of varying sizes, including miRNAs, C/D snoRNAs, and maternally expressed gene 3 (Meg3)/gene trap locus 2 (Gtl2) 12-16. Some imprinted miRNAs exist between the DLK1-DIO3 imprinted cluster Gtl2 and Mirg; as the host transcript, Rtl1as consists of seven miRNAs, while Mirg transcripts include more than 40 miRNAs 13-15, 17-19. The miRNAs in this region have strong expression levels in embryos, adult tissue organs, and placental tissues 13-15, 17-19. The DLK1-DIO3 imprinted microRNA cluster plays a profound role in the regulation of embryonic development, maintenance of mammalian stem cell pluripotency, organ building, and tumorigenesis 13-15, 17-19. Although there are studies revealing that some members of the DLK1-DIO3 imprinted microRNA cluster modulate tumor proliferation, invasion, and tumorigenicity, the in-depth molecular biological mechanisms have not yet been unveiled.

Based on the aforementioned findings, this study aimed to investigate the epigenetic mechanism underlying the inhibitory effect of anisomycin on HuOCSC activity both in vitro and in vivo. The study results confirmed that anisomycin significantly suppressed GPR137 expression levels by upregulating miR-134-3p, a member of the DLK1-DIO3 imprinted microRNA cluster. This, in turn, led to the downregulation of key proteins associated with cytoskeleton and migration, ultimately resulting in the inhibition of ex vivo HuOCSC activity.

## Materials and Methods

### Isolation and culture of primary HuOCSCs

The experiments were conducted following previously described methods 7, 18, 19. In summary, tissues were surgically isolated from four ovarian cancer patients and minced. The tissues were then subjected to digestion using 0.25% trypsin (Gibco, USA) and they were subsequently centrifuged at 1500 r/min for 5 min. CD44+/CD133+ HuOCSCs were isolated from the samples using flow cytometry.

### Cell culture and transfection

As reported previously, a final concentration of 31.8 μM (IC_50_ value) of anisomycin (Sigma-Aldrich) was applied in all of the experiments. Cells treated with the same volume of DMSO (Sigma-Aldrich) were used as a control. HuOCSCs were cultured to 70% density in cell culture dishes and then transfected with miR-134-3p oligo RNA or miR-mut oligo RNA (control) using Lipo2000 transfection reagent (GenePharma, China).

### *In vivo* tumorigenic experiment

This study used BALB/c nude mice to subcutaneously inject approximately 1 × 10^6^ logarithmically growing HuOCSCs (pre-mixed with 100 μL PBS from Gibco, USA) that had been transfected with miR-134-3p (n = 4) and miR-mut (n = 4). At the end of a 30-day period, the mice were euthanized, and the *in vivo* tumors were collected. Tumor weight and volume were measured. Tumor volume (mm^2^) was measured as (ab^2^)/2, where a and b represent the longest axis (mm) and the shortest axis (mm), respectively. Ethical approval of this animal experiment was granted by the Laboratory Animal Center of Shanghai University of Traditional Chinese Medicine.

### MTT assay

The MTT assay was conducted in line with the established protocols 7, 19. In brief, cells were cultured in 96-well plates and incubated with 10 μL of MTT reagent (Sigma, USA) at 37 °C for 3 hours at each time point. The inhibition of cell proliferation was determined by measuring the optical density (OD) at 490 nm. The inhibition of cell proliferation was calculated using the following formula: inhibition of cell proliferation (%) = (1 - OD_sample group/OD_control group) × 100%.

### Luciferase assay

All cells were seeded at 30000 cells/well in 24-well cell culture plates. Using Lipofectamine 2000 Reagent (Thermo Fisher Scientific), 400 ng of miR-134-3p or miR-mut oligo microRNA mimics and 20 ng of pGL6-GPR137-3UTR, or pGL6-GPR137-mut, or pGL6 was transfected into each group of cells, separately. At 48 h after transfection, the luciferase activity in each group of cells was examined using a dual-luciferase reporter assay system (Promega). All plasmid DNA and oligo microRNA mimics was purchased from Novobiosci (Novobiosci, China) or GenePharma (GenePharma, China).

### Flow cytometry

Approximately 2 × 10^5^ cells were harvested for centrifugation, and the precipitates were resuspended in PBS buffer and fixed in 70% ethanol in a 4 °C refrigerator overnight. The next day, the cells were digested with RNase A using a cell cycle kit (Beyotime, China) and stained with Propidium (PI). Flow analysis was performed on a flow cytometer (BD FACSAria, USA).

### Transwell assay

Cells at 70%-80% confluence were digested, centrifuged, and resuspended in a DMEM basal medium. After counting, 200 μL of cells was inoculated into the upper chamber of a 24-well Transwell at 5 × 10^4^ cells/well. In the meantime, 500 μL of DMEM complete medium was added into the lower chamber. After 24 hours of culture. After staining with 0.1% crystal violet (Beyotime, China), the cells from five fields were observed under a microscope (Leica DMI8, GER) at 10 × magnification and counted.

### Colony formation assay

DMEM medium (Gibco, USA) containing 0.6% low-melting-point agarose (Medium a) was prepared, transferred to 6-well plates (2 mL), and cooled at 4 °C to form a solid agar layer. Next, 1 mL of DMEM medium supplemented with 0.3% low-melting-point agarose (Medium b) and approximately 1 × 104 cells were combined and cooled at 4 °C to form a solid agar layer. On day 15, additional Medium b was added, and the cells were incubated at 37 °C for 1 month.

### Capillary tubule formation assay

All of the steps of northern blotting were carried out in line with the previously described protocol 19. In brief, migration assays were conducted using Matrigel-coated 6-chamber slides with a density of 2 × 10^5^ cells per chamber, containing HUVECs transfected with miR-134-3p and miR-mut. After a 6-hour incubation, the cells were captured in photographs. Four nonoverlapping regions were determined to quantify the data of branching points.

### RNA extraction and quantitative PCR (qPCR) assay

The experiments were conducted following the methods described previously 7, 18, 19. In summary, the total RNA was extracted utilizing the Trizol (EnzyArtisan, China) method, followed by the synthesis of cDNA through employment of a HyperScript III 1st Strand cDNA Synthesis Kit (EnzyArtisan, China). Ultimately, RT-qPCR was executed with the QuantStudio 6 Flex real-time PCR system (Thermo Fisher, USA) using 2 × S6 Universal SYBR qPCR Mix (EnzyArtisan, China). The primers were as follows: GPR137-F: ACCCTGTATGCCCTGCTCTTC; GPR137-R: AGGAACACCGTCTGATAGCTGAG; BASP1-F: GCAAGCTCAGCAAGAAGAAGAA; BASP1-R: CCTCGGCCTTCTTGTCTTTCTC; HSPA12B-F: CGAGAAGTTCAAGATGAAGATCCA; HSPA12B-R: CGGGCATCGTCTTTCCATTTA; FERD3L-F: AAGAGGAGTGCGAAGTGGACCA; FERD3L-R: CGCTTCCTTTCGCGGATGTTGG; OR6V1-F: GCCTTGACCTTTGTCCTCAGCT; OR6V1-R: CTGTAGCCGATGAAGACCAGTG; SSR4-F: CGCCTCTGTTTACAGTCAGCGT; SSR4-R: TGGATGTGGCTCTTCGCACTGA; XRCC3-F: TGACGTTCCAGGAGAGCTGCTT; XRCC3-R: GATGACCACCAGGCGAGCCAT; NDEL1-F: ACTAGCAGTTCGGGAAAGACAAC; NDEL1-R: AAAGAAAGTGATGCTTGGACGG; 18SrRNA-F: AGGAATTGACGGAAGGGCA; 18SrRNA-R: GGACATCTAAGGGCATCACA.

### H&E staining to measure histopathological characteristics

Paraffin tissue sections underwent dewaxing in xylene and hydration with anhydrous alcohol. Hematoxylin staining was conducted at 25 °C for 5 minutes, followed by differentiation in 1% HCl/ethanol for 30 seconds, bluing in ammonia water for 1 minute, and washing with distilled water for 5 minutes. Subsequently, eosin staining was performed for 3 minutes, followed by dehydration with an ethanol gradient. The dehydrated tissue sections were immersed in xylene twice for 4 minutes each and then sealed with neutral balsam. Finally, the sections were observed under a fluorescent microscope (Leica DMI8, GER) and photographed.

### Western blotting

The experiments were conducted following the methods described previously 7, 18, 19. In summary, the total protein that had been extracted underwent separation via 10% SDS-PAGE and was subsequently transferred onto PVDF membranes (Millipore, China). We performed sealing the PVDF membranes and incubated them with a primary antibody at 4 °C overnight. Following this, a secondary antibody was incubated at room temperature for one hour. The protein bands were then developed using an enhanced chemiluminescence assay kit (Beyotime, China). All of the antibodies were diluted at a ratio of 1:1,000. The primary antibodies utilized were anti-alpha tubulin (ab7291), anti-NDEL1 (ab 124895), anti-cytoplasmic dynein intermediate chain (ab23905), and anti-GAPDH (ab9485), all of which were procured from Abcam (Cambridge, UK). The secondary antibodies employed were HRP-linked anti-mouse IgG (#7076) and HRP-linked anti-rabbit IgG (#7074), both of which were acquired from Cell Signaling Technology (Boston, USA).

### Immunofluorescence staining

The paraffin tissue sections were subjected to baking in an oven at 80 °C for 30 minutes, followed by immersion in a xylene solution and gentle agitation on a shaker for 10 minutes, which was repeated three times. Subsequently, the sections were immersed in absolute ethanol and agitated on a shaker three times for 10 minutes each. The sections were then washed with double-distilled water for 2 minutes and exposed to a boiled antigen repair solution. After allowing the sections to cool naturally, the sections were briefly removed before reheating the solution and placing the sections back into the boiling solution. After natural cooling, the sections were removed, washed with double-distilled water for 2 minutes, and dried in an oven at 37 °C for 5 minutes. Thereafter, the sections were fully exposed to the blocking solution and incubated in an oven at 37 °C for 1 hour. Next, incubation was carried out at 4°C overnight with primary antibody, followed by three 5-minute TBST washes on a shaker and drying in an oven at 37 °C for 5 minutes. Finally, Fluoroshield™ with DAPI tissue sealer (F6057; Sigma) was dropped on the sections. The fluorescence of the sections was visualized by a fluorescence microscope (Leica DMI8, GER) and photographed. The primary antibodies utilized in this study were anti-alpha tubulin (ab7291), anti-NDEL1(ab 124895), anti-cytoplasmic dynein intermediate chain (ab23905), and anti-GAPDH (ab9485), all of which were procured from Abcam (Cambridge, UK). In addition, anti-Ki67 (PB9026) was obtained from BOSTER (Wuhan, China). The secondary antibodies employed were goat anti-rabbit IgG H&L (ab 150078) and goat anti-mouse IgG H& L (ab 150115), both of which were purchased from Abcam (Cambridge, UK) and utilized at a dilution of 1:200.

### Bioinformatics prediction and analysis

The expression profiles were obtained from GEPIA, where T (n=426) represents the patient cohort and N (n=88) represents the non-patient cohort. A series of bioinformatic analyses, including gene expression profiling analysis, pathological staging map analysis, comparative multigene analysis, and gene correlation analysis, were used to decipher the patient cohort using the GEPIA online tool 20. The Kaplan-Meier plotter online tool was utilized to visualize the survival data of 1435 ovarian cancer patients with a follow-up threshold of 250 months 21. Following this, gene ontology (GO) and pathway enrichment analyses were conducted through the PANTHER classification system 22. In addition, a protein-protein interaction network (PPI) was constructed using the STRING tool 23.

### Statistical analysis

Statistical analysis was conducted using Graph Pad Prism 9. The data were acquired from experiments conducted in triplicate and presented as means ±SDs. Student's t test and one-way ANOVA (with multiple comparisons) were utilized for comparisons in the presence of normal distribution, while Mann-Whitney test and Kruskal-Wallis (with multiple comparisons) were employed when the data did not adhere to normal distribution.

## Results

### Anisomycin drives miR-134-3p to suppress GPR137 expression

**Figure 1:**
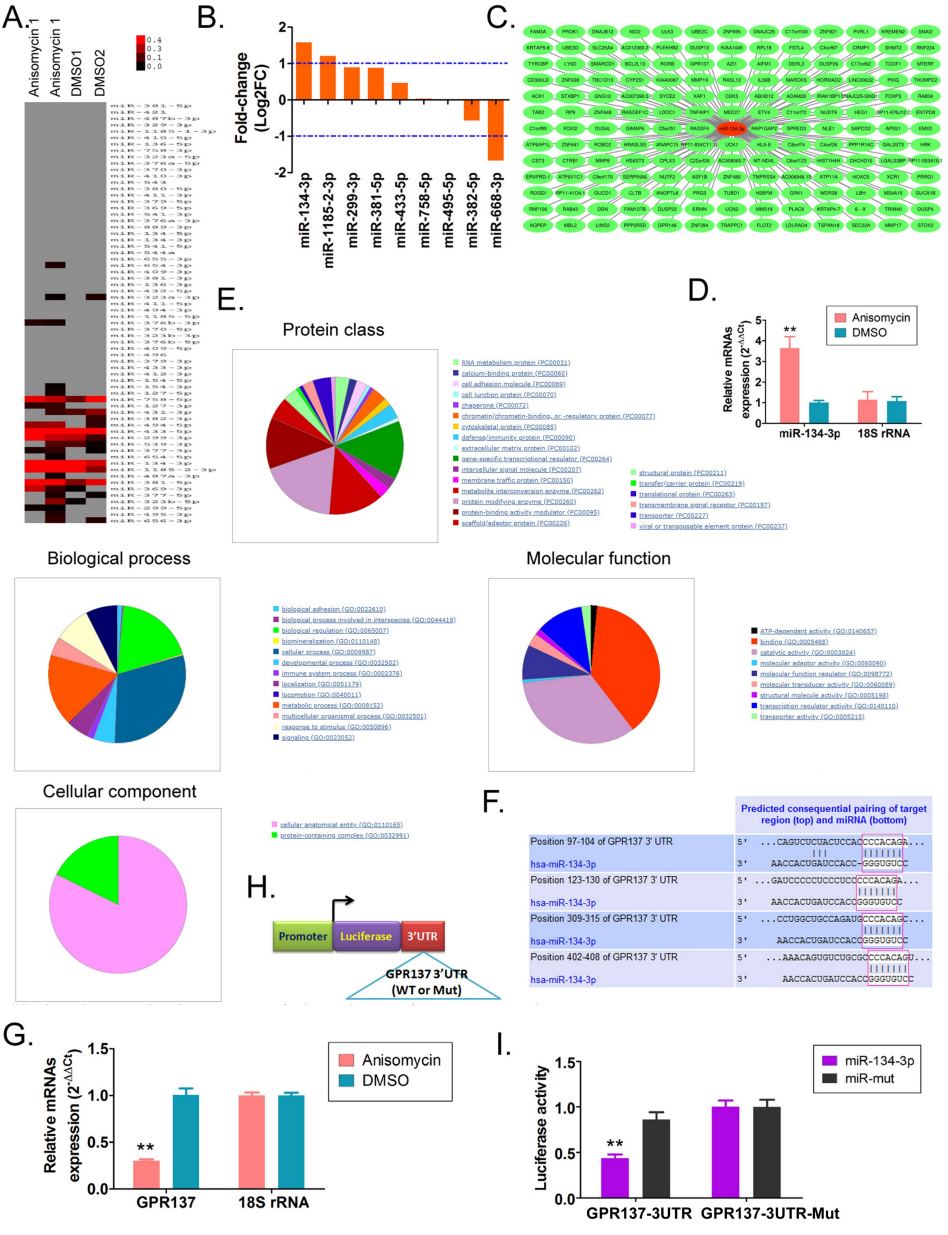
** Anisomycin downregulates GPR137 expression by targeting miR-134-3p.** (A) The results of cDNA microArray assay. (B) The expression of DLK1-DIO3 imprinted microRNA cluster members. (C) Prediction of the candidate target genes. (D) The endogenous expression levels of miR-134-3p in anisomycin-treated HuOCSCs (n = 3) detected by RT-qPCR assay; ** P < 0.01 vs. DMSO. *t* test. (E) Prediction of biological effects of candidate genes targeted by miR-134-3p. (F) Complementary pairing characteristics of miR-134-3p and the candidate target gene GPR137. (G) The expression level of GPR137 in anisomycin-treated HuOCSCs (n = 3) measured by RT-qPCR; ** P < 0.01 vs. DMSO. *t* test. (H) Construction of plasmids containing 3'-UTR-specific locus of the GPR137 gene. (I) Luciferase reporter gene assay (n = 3); ** P < 0.01 vs. miR-mut. *t* test.

The cDNA microArray assay showed that multiple members of DLK1-DIO3 imprinted microRNA cluster (miR-134-3p, miR-1185-2-3p, miR-299-3p, miR-381-5p, miR-433-5p) had significantly higher expression in anisomycin-treated HuOCSCs than in the control group (Figure 1A). Among these members, miR-134-3p was the most differentially expressed between the two groups (Figure 1B). By searching the TargetScan database (http://www.targetscan.org), we identified 169 genes that were the candidate target genes for miR-134-3p (Figure 1C). qPCR pointed out that HuOCSCs treated by anisomycin had significantly higher expression of endogenous miR-134-3p (3.64±0.56) than the control group (1.01±0.09) (Figure 1D). Subsequently, GO analysis indicated that miR-134-3p-targeted regulatory candidate genes corresponded to different biological effects, such as protein-modifying enzyme (Protein class), metabolite interconversion enzyme (Protein class), catalytic activity (Molecular function), cellular process (Biological process), and cellular anatomical entity (Cellular component) (Figure 1E). Sequence alignment through the database revealed that miR-134-3p and the candidate target gene GPR137 had complementary pairing features at multiple sites of the 3'-UTR, and all of these sites had a full complementary pairing of seven nucleotides (5'-CCCACAG-3') (Figure 1F). Subsequently, qPCR results also supported that the endogenous expression level of GPR137 was markedly lower in anisomycin-treated HuOCSCs (0.30±0.02) compared with the control group (1.00±0.07) (Figure 1G). Further, we constructed an expression plasmid carrying the 3'-UTR-specific locus of the GPR137 gene (Figure 1H). Luciferase report assay results showed an apparent reduction in the luciferase activity of wild-type GPR137 gene carrying the 3'-UTR-specific locus induced by miR-134-3p (Figure 1I). Therefore, the above findings suggested that GPR137 was one of the genes specifically targeted for regulation by miR-134-3p. Additionally, anisomycin suppressed GPR137 expression by driving miR-134-3p upregulation.

### miR-134-3p overexpression significantly inhibits the *in vitro* and *in vivo* activities of HuOCSCs

**Figure 2:**
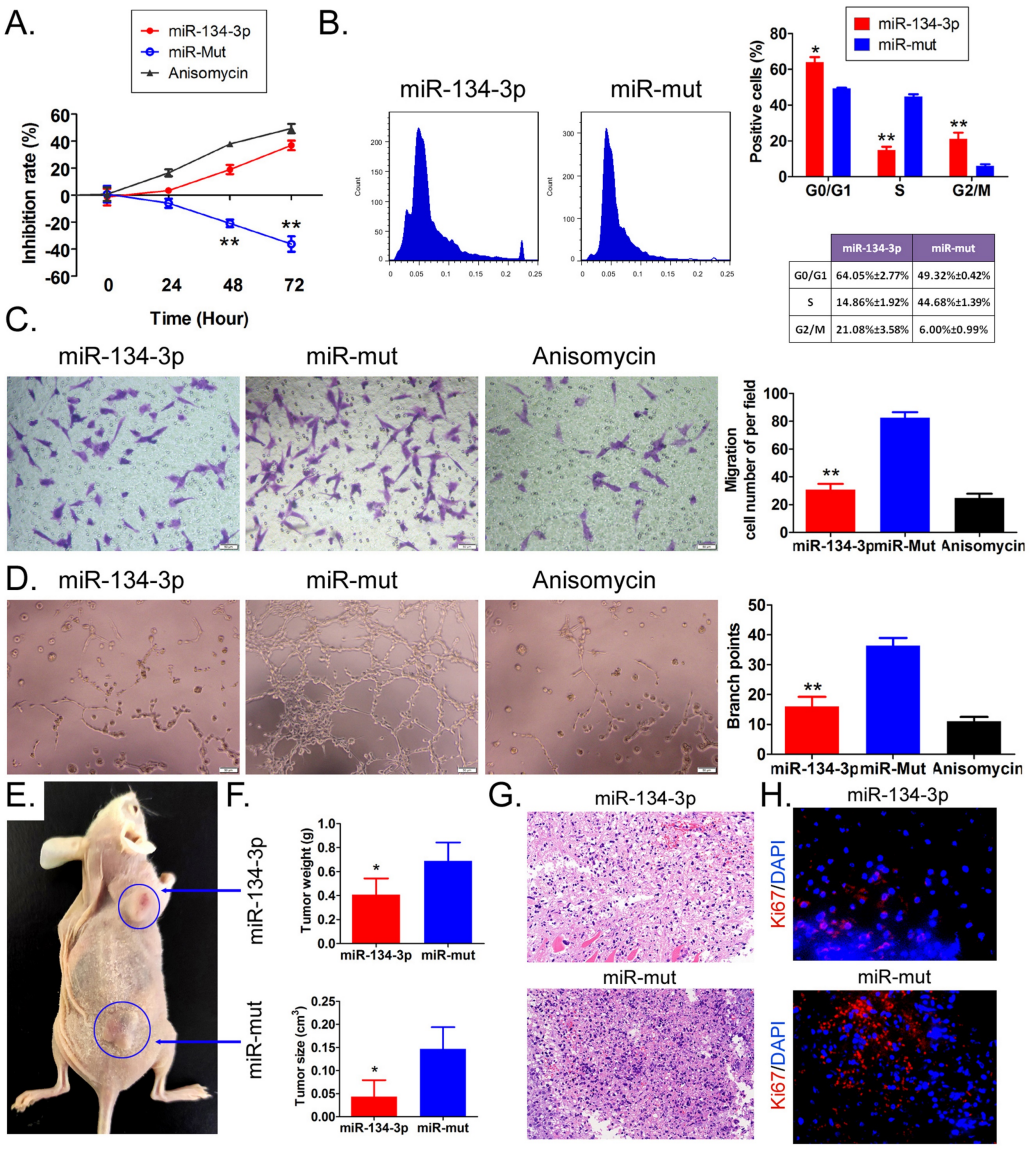
** miR-134-3p overexpression inhibits HuOCSC activity both *in vitro* and *in vivo*.** (A) Proliferation inhibition rate assay (n = 3). ** P < 0.01 vs. miR-mut.* t* test. (B) Cell cycle assay (n = 3). * P < 0.05 vs. miR-mut; ** P < 0.01 vs. miR-mut. *t* test. (C) Transwell assay of migration ability of HuOCSCs (n = 3). ** P < 0.01 vs. miR-mut. *t* test. (D) Angiogenesis assay to detect the ability of formation of tubular 3D structures (n = 3). ** P < 0.01 vs. miR-mut. *t* test. (E) *In vitro* graft tumor assay. * P < 0.05 vs. miR-mut. *t* test. (F) The weight and volume of graft tumors. * P < 0.05 vs. miR-mut. *t* test. (G) HE staining (2× magnification). (H) Immunofluorescence labeling of Ki67 (400× magnification).

The results of the MTT assay indicated a significant increase in the rate of proliferation inhibition of HuOCSCs treated with miR-134-3p in a time-dependent manner, compared with the control group consisting of miR-mut-transfected HuOCSCs. According to flow cytometry assay results, miR-134-3p-transfected HuOCSCs showed larger amounts of G0/G1 and G2/M phase cells compared with the control group, while they had significantly lower proportions of S phase cells (Figure 2B). Moreover, transwell assay revealed that miR-134-3p-transfected HuOCSCs had significantly fewer migrated cells than the control group (Figure 2C). Additionally, angiogenesis assay in the outer matrix of HUVECs showed that miR-134-3p overexpression significantly hindered HUVECs to form tubular 3D structures in Matrigel (Figure 2D).

The results of *in vivo* experiments revealed that both miR-134-3p-transfected HuOCSCs and miR-mut-transfected HuOCSCs (control group) were able to form subcutaneous tumors in nude mice (Figure 2E). However, the tumors that originated from miR-134-3p-transfected HuOCSCs were strikingly smaller in both volume and weight than those from the control group (Figure 2F). H&E staining suggested that although the tumor tissues from both groups were consistent with ovarian clear cell carcinoma characteristics, the tumor tissues derived from miR-134-3p-transfected HuOCSCs had markedly lower levels of nuclear division phase, cell naivety, and tumor malignancy than the control group (Figure 2G). Meanwhile, the outcomes of tissue immunofluorescence staining demonstrated a noteworthy decrease in the expression of cytokinesis Ki67 in the tumor tissues derived from HuOCSCs transfected with miR-134-3p, relative to the control cohort (Figure 2G). Therefore, the outcomes of both *in vitro* and *in vivo* experiments demonstrated that the upregulation of miR-134-3p effectively impeded the proliferation, migration, and tumorigenic potential of HuOCSCs.

### miR-134-3p overexpression significantly downregulates the expression of GPR137 and related proteins

**Figure 3:**
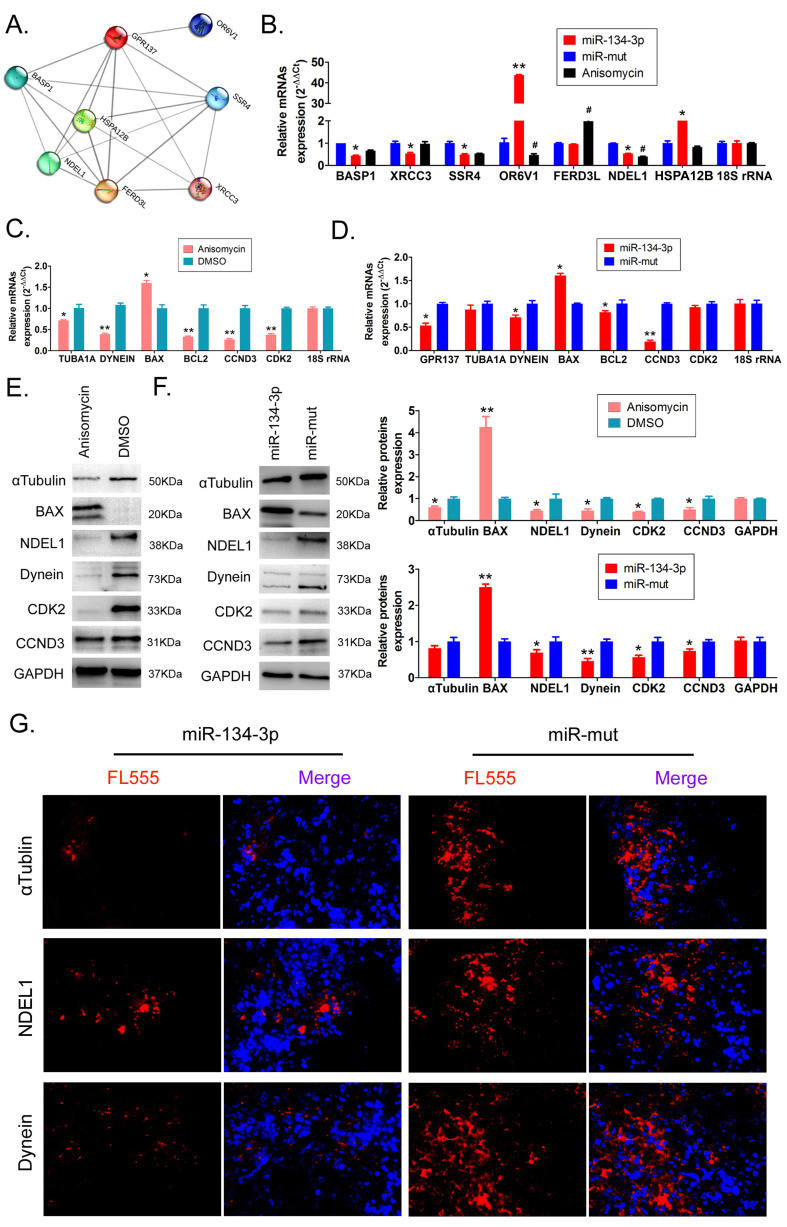
** miR-134-3p overexpression regulates GPR137 expression levels and its associated proteins.** (A) PPI network of GPR137 and its associated proteins. (B) RT-qPCR assay of GPR137 and its related gene expression (n = 3). * P < 0.05 vs. miR-mut, ** P < 0.01 vs. miR-mut. *t* test. (C) Detection of the GPR137/NDEL1/DYNEIN/TUBA1A axis and cell cycle factor and apoptosis gene expression in anisomycin-treated HuOCSCs by RT-qPCR (n = 3). * P < 0.05 vs. DMSO, ** P<0.01 vs. DMSO. *t* test. (D) The gene expression of the GPR137/NDEL1/DYNEIN/TUBA1A axis in the miR-134-3p overexpression group (n = 3). * P < 0.05 vs. DMSO, ** P < 0.01 vs. DMSO. *t* test. (E) Western blot for measuring the protein expression of the GPR137/NDEL1/DYNEIN/TUBA1A axis and cell cycle factor and apoptosis gene protein expression in the anisomycin-treated HuOCSCs group (n = 3). * P < 0.05 vs. DMSO, ** P < 0.01 vs. DMSO. *t* test. (F) Western blot for measuring the protein expression of the GPR137/NDEL1/DYNEIN/TUBA1A axis and cell cycle factor and apoptosis gene protein expression in the miR-134-3p overexpression group (n = 3). * P < 0.05 vs. miR-mut, ** P < 0.01 vs. miR-mut. *t* test. (G) Immunofluorescence labeling of the NDEL1/DYNEIN/TUBA1A axis-related proteins (400× magnification).

The results of the bioinformatics prediction showed that several proteins (OR6V1, SSR4, XRCC3, BASP1, FERD3L, HSPA12B, and NDEL1) interacted with GPR137 (Figure 3A). qPCR results indicated that SSR4, XRCC3, BASP1, and NDEL1 expression levels were significantly downregulated in HuOCSCs overexpressing miR-134-3p, which was accordant with the results in anisomycin-treated HuOCSCs (Figure 3B, Supplementary data Table S1). Based on the results of previous research 24, 25, we chose to perform an in-depth study of the GPR137/NDEL1/DYNEIN/TUBA1A axis. The qPCR findings indicated a significant decrease in the expression levels of DYNEIN, TUBA1A, cell cycle factors (CCDN3, CDK2), and antiapoptotic gene BCL2 in the anisomycin-treated group compared with the control group. Conversely, the proapoptotic gene BAX demonstrated elevated expression (Figure 3C, Supplementary data Table S2). Concurrently, the qPCR outcomes demonstrated that the group treated with anisomycin exhibited notably reduced expression levels of GPR137, DYNEIN, CCDN3, and BCL2, and displayed elevated BAX expression level, compared with the control group (Figure 3D, Supplementary data Table S3).

The results of western blot analysis indicated a noteworthy reduction in the expression levels of GPR137, NDEL1, DYNEIN, and TUBA1A in both the anisomycin-treated and the miR-137-3p overexpression groups, compared with the control group (Figure 3E, 3F, Supplementary data Figure S1, Table S4, S5). Moreover, the results were corroborated by immunofluorescence staining, which demonstrated a statistically significant reduction in the expression levels of NDEL1, DYNEIN, and TUBA1A in the tumor tissues obtained from miR-134-3p-treated HuOCSCs compared with those derived from miR-mut-transfected HuOCSCs (Figure 3G, Supplementary data Figure S2). The findings presented herein provide evidence that the upregulation of miR-134-3p leads to a significant decrease in the expression of GPR137 and its related proteins, namely NDEL1, DYNEIN, and TUBA1A.

### The expression levels of GPR137, NDEL1, DYNC1H1, and TUBA1A are closely associated with the advancement and prognosis of ovarian cancer

**Figure 4:**
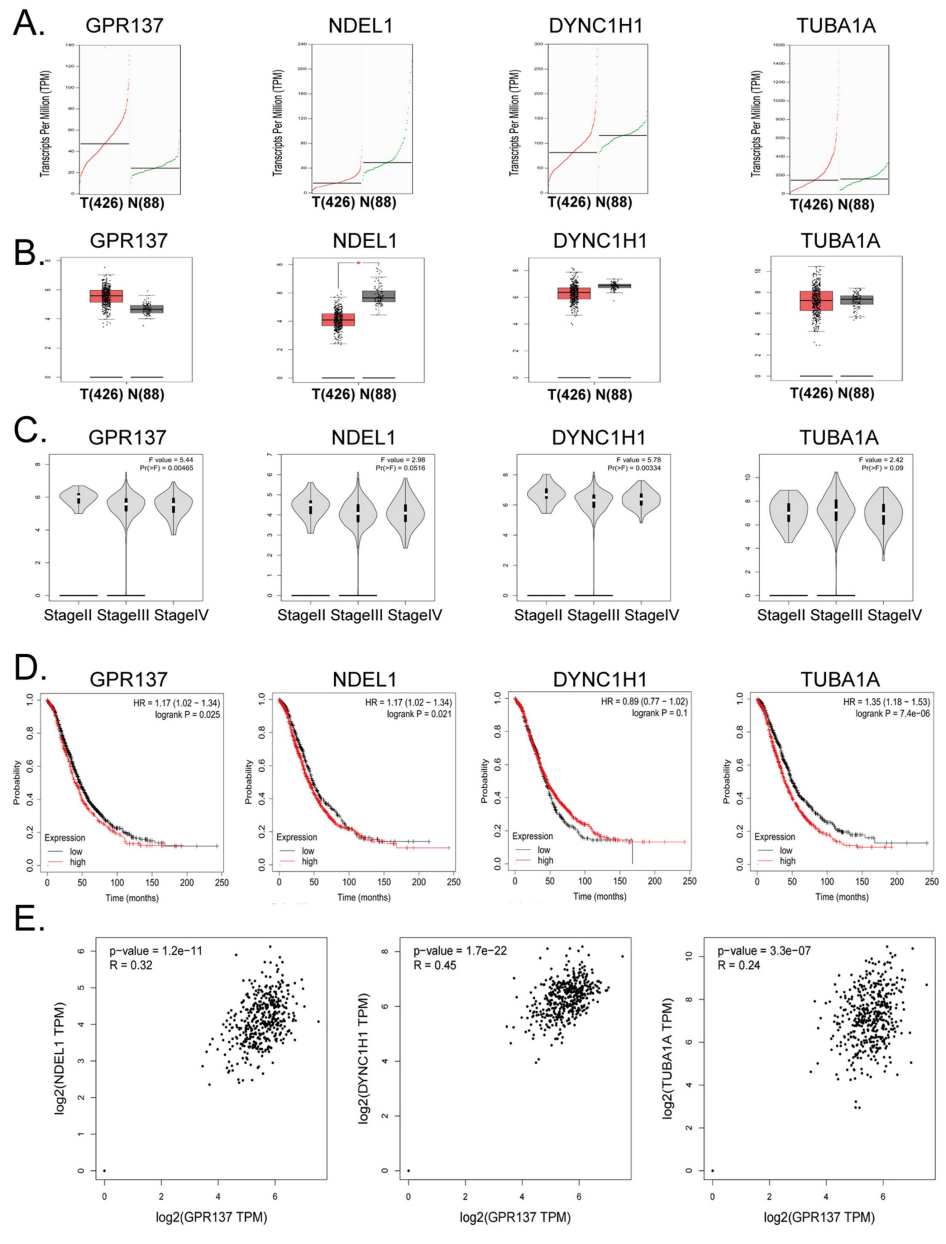
** Bioinformatics prediction analysis of GPR137, NDEL1, DYNC1H1, and TUBA1A.** (A) Transcriptional expression levels of GPR137, NDEL1, DYNC1H1, and TUBA1A genes. (B) The expression levels of GPR137, NDEL1, DYNC1H1, and TUBA1A genes. (C) GPR137, NDEL1, DYNC1H1, and TUBA1A gene expression at different pathological stage levels. (D) Prognostic analysis of GPR137, NDEL1, DYNC1H1, and TUBA1A. (E) Correlation analysis of GPR137 with NDEL1, DYNC1H1, and TUBA1A.

Subsequent to the aforementioned observations, we undertook additional investigations to examine the inherent correlation between the genes GPR137, NDEL1, DYNC1H1, and TUBA1A and the advancement and prognosis of ovarian cancer. Examination of the cDNA expression profile disclosed elevated GPR137 expression in the neoplastic tissues of ovarian cancer patients (Figure 4A, B). Analysis of pathological stage indicated differential expression levels of GPR137 and DYNC1H1 across varying stages of ovarian cancer (Figure 4C).

The findings of the study indicate that ovarian cancer patients with elevated expression of GPR137, NDEL1, and TUBA1A genes experienced a statistically significant increase in survival time, as demonstrated by Kaplan-Meier survival analysis (Figure 4D). These results suggest a potential association between the expression levels of these genes and the development of ovarian cancer, as well as the survival of patients. Further, multiple gene correlation analyses revealed a significant positive linear relationship between GPR137 expression in ovarian cancer tissues and the expression levels of NDEL1, DYNC1H1, and TUBA1A (Figure 4E). Thus, the findings of the analysis indicate a significant correlation of the expression levels of GPR137, NDEL1, DYNC1H1, and TUBA1A with the advancement and prognosis of ovarian cancer.

## Discussion

A growing number of studies have pointed out that besides its antibiotic effect, anisomycin is able to effectively hinder the proliferation, migration, and tumorigenicity of a variety of tumors 8-10, 19, 26. Initial studies on the target of anisomycin focused on the regulation of protein activity 26. Previous studies have shown that anisomycin is a potent inhibitor of protein and DNA synthesis via interfering with the peptidyl transferase 80 ribosome system 9, 10, 26. There is also evidence that anisomycin is an agonist of c-Jun N-terminal kinase (JNK), which enhances the level of JNK protein phosphorylation 8-10, 26.

As research progressed, it has been successively reported that anisomycin causes aging degeneration in neuronal cells, mainly through repressing methylation transferase activity, and thereby leads to demethylation modification in the promoter regions of β-site APP-cleaving enzyme 1 (BACE1), amyloid-β precursor protein (APP), and presenilin 1 (PS1) genes, and induces high expression of the above genes 26-28. By contrast, increased expression of histone acetyltransferase p300/CREB results in acetylation modification of histone H3 and promotes the transcription and activation of APP, BACE1, and PS1 genes, which eventually releases toxic amyloid proteins, leading to neuronal aging, degeneration, and necrosis 26-28. Therefore, anisomycin is not a single-target drug. Additionally, a recent report has indicated that anisomycin induces ferroptosis in hepatocellular carcinoma cells by promoting phosphorylation of histone H3 on serine 10 (p-H3S10) via p38 MAPK 11. These studies suggest that the function of anisomycin is much more complicated than we thought, and its effects on cellular physiological and biochemical actions are likely closely linked to epigenetic alterations.

In our previous studies, we have hypothesized that anisomycin has a regulatory effect on epigenetic modifications in tumor cells. We found that anisomycin, on the one hand, was able to enhance the stability and activity of BACE1 and APP mRNAs by activating the expression of long noncoding RNA β-site APP cleaving enzyme 1 antisense strand (lncRNA-BACE1-AS), and finally led to massive expression of toxic amyloid Aβ1-42, which can cause the death of OCSCs 11. On the other hand, it has also been confirmed that anisomycin hinders the activation of the Notch 1 pathway downstream by attenuating the sponge effect of the lncRNA-Meg3/miR-421/PDGFRA axis, ultimately resulting in the suppression of OCSC proliferation, angiogenesis, and invasion 19. These studies have been the first to validate that anisomycin has a regulatory effect on epigenetics, especially on noncoding RNAs. Following this direction, we analyzed the expression of the DLK1-DIO3 imprinted microRNA cluster members in the anisomycin-treated HuOCSCs (we selected this target mainly because the DLK1-DIO3 imprinted microRNA cluster has a significant regulatory role in tumor cell activity 12-16). The results showed that anisomycin was indeed able to alter the expression levels of several DLK1-DIO3 imprinted microRNA cluster members, with miR-134-3p being the most significantly upregulated member. Subsequently, *in vitro* and *in vivo* analysis further verified our speculation that independent overexpression of exogenous miR-134-3p significantly impairs the proliferation, migration, clone formation, angiogenesis, and tumorigenicity of OCSCs. The phenotypic changes were consistent with those exhibited by anisomycin-treated ovarian stem cells, which coincided with our previous study. We found that normal ovarian cancer cells (CD44-/CD133-) had high expression levels, while miR-134-3p expression was extremely low in HuOCSCs 18, suggesting that miR-134-3p is inversely correlated with the malignancy of ovarian cancer.

**Figure 5:**
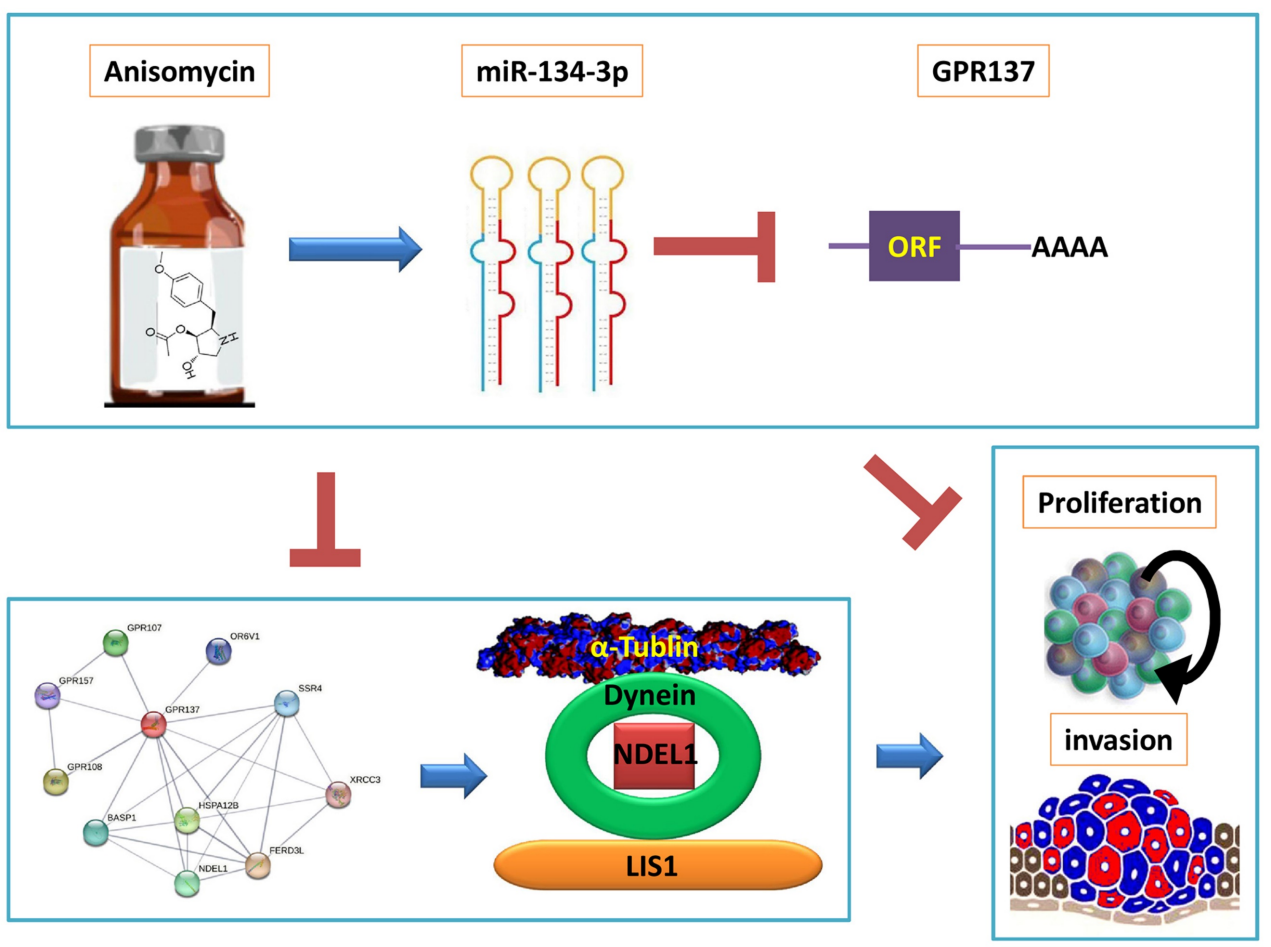
** miR-134-3p mediates the GPR137/NDEL1/DYNEIN/TUBA1A axis in HuOCSCs.** The diagram illustrates that anisomycin upregulates the DLK1-DIO3 family miR-3p-134 and inhibits the expression of the GPR137/NDEL1/DYNEIN/TUBA1A axis, thereby leading to the suppression of HuOCSCs activity.

Subsequently, we predicted the negative regulatory targets of miR-134-3p using bioinformatic analysis and protein network interactions. We observed that the expression of miR-134-3p significantly downregulated the expression level of GPR137. Moreover, the PPI prediction results suggested that GPR137 interacted with NDEL1, DYNC1H1, and TUBA1A proteins. It has been reported that NDEL1, DYNC1H1, and TUBA1A proteins are closely linked to the skeleton formation and cell migration of tumor cells, and suppressing the above proteins leads to the loss of intrinsic morphology and invasive ability of tumor cells, and even cell deformation and death 24, 25. We also discovered that overexpression of miR-134-3p resulted in a significantly decreased expression of NDEL1, DYNC1H1, and TUBA1A proteins downstream of GPR137. Moreover, by examining clinical data, we found that the expression of four genes, GPR137, NDEL1, DYNC1H1, and TUBA1A, differed significantly between tumor tissues and normal tissues, and their expression levels were significantly associated with poor prognosis of ovarian cancer patients. These findings provide theoretical support for exploring effective therapeutic agents for ovarian cancer in the future (Figure 5).

In summary, we revealed that anisomycin could regulate miR-134-3p, a member of DLK1-DIO3 imprinted microRNA cluster, to inhibit OCSC activity by suppressing the target gene GPR137 and downregulating the expression of NDEL1, DYNC1H1, and TUBA1A proteins.

## Supplementary Material

Supplementary figures and tables.

## Abbreviations

HuOCSCs: Human ovarian cancer tumor stem cells
MAPK: Mitogen-activated protein kinase
Dlk1: Delta-like homologue 1
Dio3: type 3 deiodinase
Rtl1: Retrotransposon-like gene 1
Meg3: Maternally expressed gene 3
Gtl2: Gene trap locus 2
RT-qPCR: RNA extraction and quantitative PCR
JNK: c-Jun N-terminal kinase
BACE1: β-site APP-cleaving enzyme 1
APP: amyloid-β precursor protein
p-H3S10: phosphorylation of histone H3 on serine 10
