# Supplementary material for: miR-134-3p driven by anisomycin impairs ovarian cancer stem cell activity through inhibiting GPR137 expression
Source: J Cancer. 2023 Oct 16;14(18):3404–15. doi: 10.7150/jca.87692 (PMC10647200; doi:10.7150/jca.87692)
Supplement: Supplementary file 1 — Supplementary figures and tables. [file jcav14p3404s1.pdf]

Mir-134-3p Driven by Anisomycin Impairs Ovarian Cancer Stem Cell Activity through Inhibiting GPR137 Expression

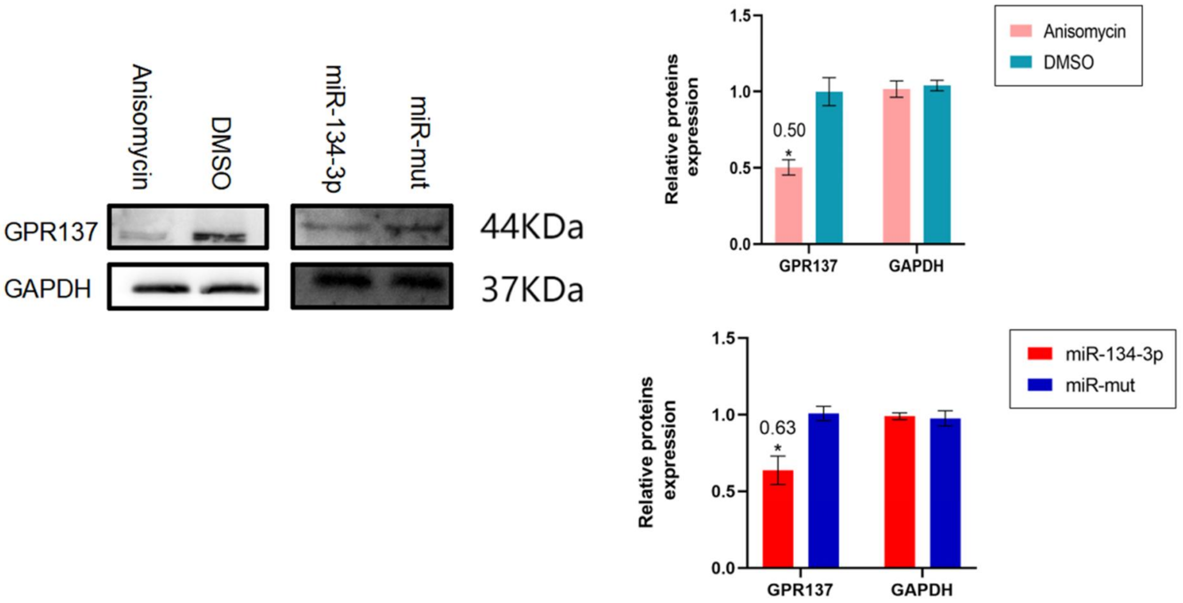

Figure S1 The results of western blot assay.

*** P < 0.05 vs. DMSO, * P < 0.05 vs. miR-mut.**

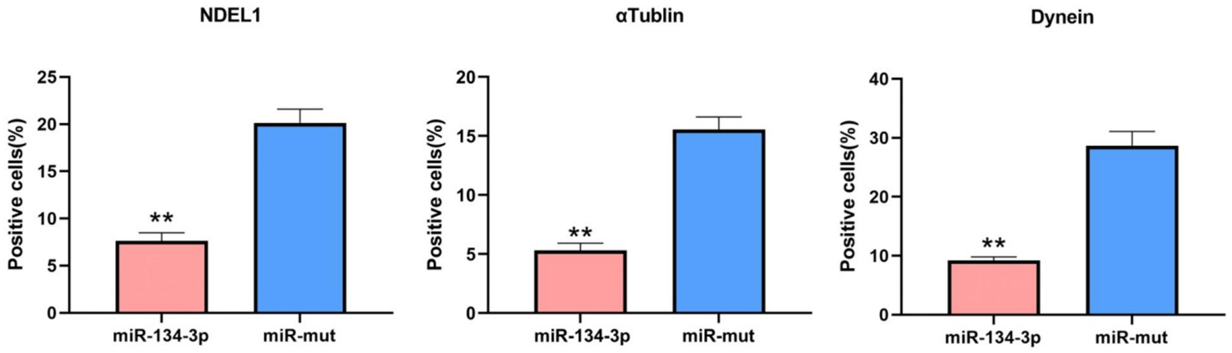

Figure S2 The statistical analysis results of Immunofluorescence staining.

**** P < 0.01 vs. miR-mut, n = 3.**

Table S1 The results of qPCR.

Gene	miR-mut	miR-134-3p	Anisomycin
**BASP1**	1.00±0.00	0.44±0.02	0.66±0.03
**XRCC3**	1.01±0.08	0.53±0.05	0.98±0.09
**SSR4**	1.00±0.07	0.49±0.04	0.54±0.01
**OR6V1**	1.04±0.18	43.70±0.23	0.47±0.06
**FERD3L**	1.00±0.02	0.96±0.00	1.99±0.05
**NDEL1**	1.00±0.00	0.53±0.01	0.39±0.02
**HSP12B**	1.01±0.10	19.69±0.19	0.83±0.04
**18S rRNA**	1.01±0.08	1.01±0.09	1.00±0.02

Table S2 The results of qPCR.

Gene	Anisomycin	DMSO
**TUBA1A**	0.71±0.02	1.01±0.09
**DYNEIN**	0.39±0.02	1.08±0.04
**BAX**	1.60±0.06	1.01±0.08
**BCL2**	0.33±0.02	1.01±0.07
**CCND3**	0.26±0.02	1.00±0.07
**CDK2**	0.38±0.02	1.00±0.03
**18S rRNA**	1.00±0.03	1.00±0.03

Table S3 The results of qPCR.

Gene	miR-134-3p	miR-mut
**GPR137**	0.54±0.05	1.00±0.03
**TUBA1A**	0.88±0.10	1.00±0.05
**DYNEIN**	0.71±0.05	1.00±0.06
**BAX**	1.60±0.05	1.00±0.02
**BCL2**	0.82±0.04	1.01±0.08
**CCND3**	0.19±0.03	1.00±0.02
**CDK2**	0.93±0.04	1.00±0.04
**18S rRNA**	1.01±0.09	1.01±0.07

Table S4 The results of Western blotting.

Proteins	Anisomycin	DMSO
α Tubulini	0.60±0.06	1.00±0.08
BAX	4.26±0.48	1.00±0.05
NDEL1	0.45±0.06	1.00±0.21
Dynein	0.46±0.08	1.00±0.04
CDK2	0.40±0.03	1.00±0.02
CCND3	0.50±0.09	1.00±0.11
GAPDH	1.00±0.04	1.00±0.01

Table S5 The results of Western blotting.

Proteins	miR-134-3p	miR-mut
α Tubulin3	0.81±0.07	1.00±0.12
BAX	2.51±0.08	1.00±0.08
NDEL1	0.69±0.08	1.00±0.13
Dynein	0.46±0.07	1.00±0.07
CDK2	0.57±0.06	1.00±0.12
CCND3	0.74±0.06	1.00±0.05
GAPDH	1.03±0.09	1.00±0.12
